# Congenital Abdominal Wall Defects: Staged closure by Dual Mesh

**Published:** 2016-01-01

**Authors:** Kirsten Risby, Marianne Skytte Jakobsen, Niels Qvist

**Affiliations:** 1 Hans Christian Andersen Children´s Hospital, Odense University Hospital, Denmark; 2 Department of Pediatrics, Kolding Hospital, Denmark; 3 Department of surgery A (Pediatric Surgery), Odense University Hospital, Denmark

**Keywords:** Gastroschisis, Omphalocele, Mesh, Gore DualMesh

## Abstract

**Objective:** To evaluate the clinical utility of GORE® DUALMESH (GDM) in the staged closure of large congenital abdominal wall defects. **Materials and Methods:** Data of patients with congenital abdominal wall defects managed with GDM was analyzed for outcome regarding complete fascial closure; mesh related complications; and post-discharge gastrointestinal surgery. **Results:** GDM was placed in 34 (gastroschisis=27, omphalocele=7) patients during the study period. Complete closure of the fascia was obtained in one patient with omphalocele and in 22 patients with gastroschisis. Mesh related surgical complications were seen in five (15%) children: four had detachment of the mesh and one patient developed abdominal compartment syndrome. Mesh related clinical infection was observed in five children. In hospital mortality occurred in four cases (2 gastroschisis and 2 omphalocele) and was not procedure-related. Of the 30 children discharged, 28 (82%) were still alive. At follow-up, three patients (10%) were operated for a minor ventral hernia and 4 children were operated (laparotomy and adhesionolysis) for adhesive intestinal obstruction. **Conclusion:** Staged closure with GDM is a safe alternative when primary fascial closure is difficult.

## INTRODUCTION

In infants with congenital abdominal wall defect, the ultimate surgical goals are to reduce the herniated viscera into the abdomen with a final complete closure of the fascia and skin to create a solid abdominal wall with an acceptable cosmetic result. It is essential that this is done without risking abdominal compartment syndrome or tissue damage [1, 2]. In up to 79% [3] of the infants, primary closure of the fascia is impossible; where several other techniques have been described including Silastic chimney (silo) construction with staged reduction of the viscera and secondary closure of the abdomen [4], prosthetic patch defect closure [5, 6], skin flap closure [7] or topical therapy with epithelialization [8] followed by secondary ventral hernia repair.

It is not known which type of treatment involves the lowest risk of complications and the highest success rate of secondary closure of the fascia, especially in those children with large defects and evisceration. The aim of this retrospective study was to evaluate the clinical utility of GDM in the staged secondary closure of large congenital abdominal wall defects. The primary outcomes under evaluation were complete fascial closure; the nature and frequency of mesh related complications; and post-discharge gastrointestinal surgery.

## MATERIALS AND METHODS

A review of medical record of children, with a diagnosis of gastroschisis (DQ79.3) or omphalocele (DQ79.2) in whom the GDM (GORE, Flagstaff, Arizona, USA) was used for surgical repair during the period from 1 January 1996 to 1 September 2014 in a tertiary center, was done. The mesh was applied to the patients where it was impossible to reduce more than two thirds of the estimated volume of the herniated organs or with a defect > 5 cm.

**Technique:**

After the umbilical vessels had been ligated, the mesh was sutured to the fascial edges of the defect to create a silo. Abdominal tension was evaluated daily by abdominal palpation and general clinical condition. When further reduction of the organs was considered possible (respiratory frequency < 25/min, O2 saturation >96% by pulse oximetry and if on ventilator an inspiratory pressure < 10 mmHg and with a fraction of O2 < 0.4, the mesh was reopened at top midline and any adhesions between intestines and abdominal wall were freed to further reduce the organs into the abdominal cavity. Then the mesh was reduced correspondingly and closed by a running suture (Fig. 1) under maximal traction without compromising ventilation (max inspiration pressure below 15 mm Hg). This was continued until outcome of closure was reached. 

**Figure:**
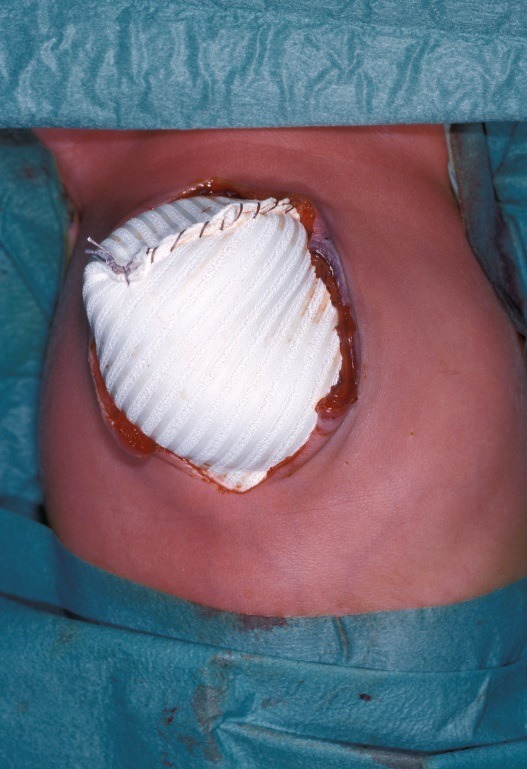
Figure 1: Top view of inserted GDM.

Data extracted included prenatal diagnosis, type of defect, type and place of birth, sex, gestational age, birth weight, additional congenital anomalies, numbers of operation, numbers of mesh adjustments, and the age at complete fascial closure. Postoperative data reviewed were complications including detachment of the mesh, wound infections, and post-discharge gastrointestinal surgery. The study was approved by the Regional committee of Biomedical Ethics (Project-ID S-20120215).

## RESULTS

During the study period, a total of 181 infants underwent repair of a congenital abdominal wall defect; 104 with gastroschisis and 77 with omphalocele. A mesh was placed in 34 patients (gastroschisis=27, omphalocele=7). A summary of clinical background data on the children operated with mesh is presented in Table l.

**Figure:**
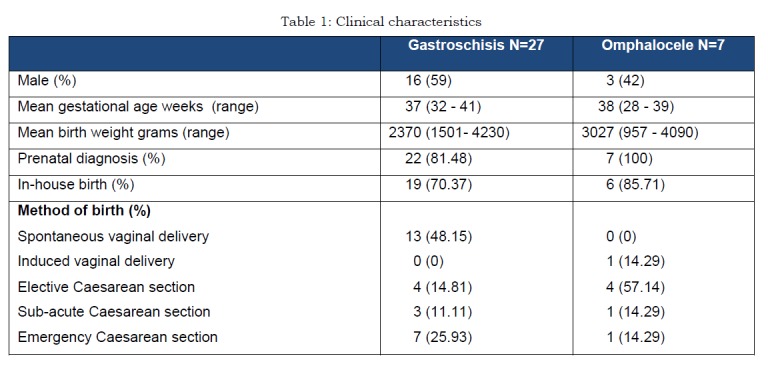
Table 1: Clinical characteristics

**Mortality and concomitant congenital malformations:**

Of the 34 children operated with mesh two children (one with gastroschisis and one with omphalocele) died with the mesh in situ before closure was possible. Thus 32 completed treatment and 28 of these children (82%) were still alive (September 2014) (mean age 9.5 years, range 0-18). The total mortality was three children from the gastroschisis group (11%) and three from the omphalocele group (43%) (Table 3). In the six fatality cases five had concomitant congenital malformations. Among those who were still alive, only two had concomitant congenital malformations. 

**Figure:**
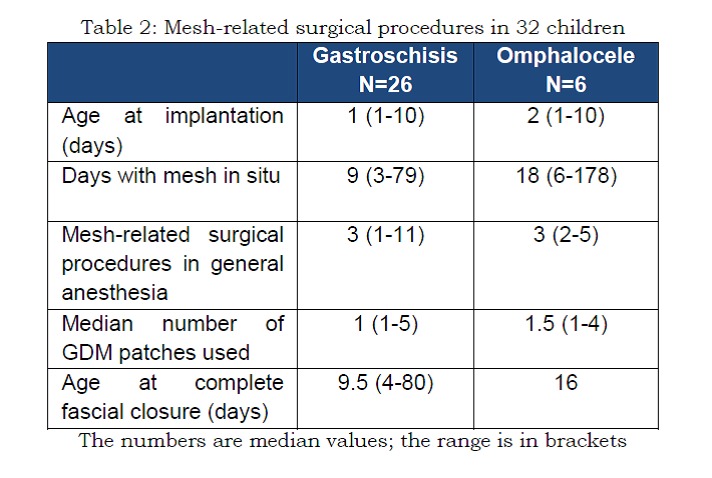
Table 2: Mesh-related surgical procedures in 32 children. The numbers are median values; the range is in brackets.

**Mesh-related surgical procedures:**

In 91% of the cases, the mesh was implanted as the primary procedure; in the rest, it was secondary to a failed Silastic silo. The age of the child at implant of mesh ranged between 1 and 10 days (Table 2). On average three mesh-related procedures were performed in these patients with a range of 1-11 interventions for sequential reduction of eviscerated contents. Removal of the mesh was done within 11 days in 50% of the children, within 22 days in 75% and within 44 days in 90% of the children (Fig. 2).

**Figure:**
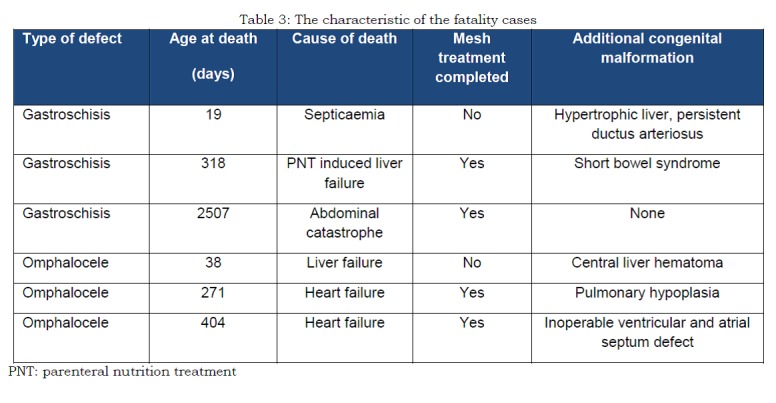
Table 3: The characteristic of the fatality cases. PNT: parenteral nutrition treatment.

**Figure:**
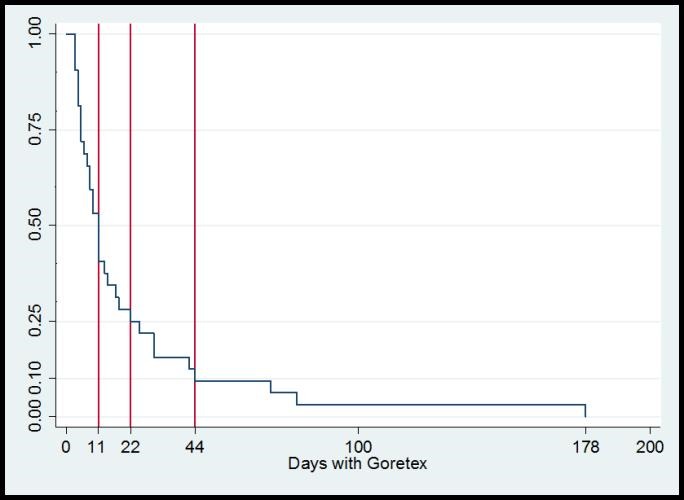
Figure 2: The duration of the treatment of GDM. In 50% of the children the mesh was removed within 11 days, in 75% within 22 days and in 90% within 44 days

**Abdominal defect closure:**

Among the children who completed surgical treatment a complete closure of the fascia was obtained in only one (16th day) out of six children with omphalocele and in 22 out of 26 (on average 9.5 days) with gastroschisis (Table 2). In the remaining nine children where fascial closure was not possible, the mesh was left in situ and removed after a period of 14 days to six months. In five children epithelialization of defect occurred, and in four skin grafts were needed.

**Complications related to insertion of the mesh: **

Surgical mesh-related complications were seen in five (15%) children. In four of these the mesh detached requiring re-suturing or reimplantation of a new mesh. The fifth child developed abdominal compartment syndrome requiring temporary loosening of the mesh. Clinical infection related to the mesh defined as fever, redness of the wound and elevated serum C-reactive protein levels was observed in five (15%) children. Four of these were treated by systemic antibiotics, and in one patient a premature mesh removal was chosen. Suture granulomas were resected twice in one child, at age 3.5 and 8 years old, respectively. No mesh-related complication caused death. 

**Post-discharge gastrointestinal surgery:**

One child was later diagnosed with biliary atresia and Kasai procedure done at 80th day of life. Three patients (10%) were operated on for a minor ventral hernia. During the observation period laparotomy with adhesionolysis for adhesive bowel obstruction in two children (7%), aged 5 months and 5 years respectively, and laparotomy for a chronic ileus condition in three (10%): one due to a primary unrecognized sickle-shaped jejunal atresia and two due to peritoneal adherences. Four children (13%) have been admitted to hospital for a conservative treatment of symptoms of ileus at the age of two, five, eight, and fifteen years, respectively.

## DISCUSSION

The present study showed a low rate of secondary closure of the fascia in children with omphalocele compared with gastroschisis defects. A comparison with other studies is difficult as most studies with staged closure include large as well as minor defects. The procedure-related complications were minimal, but a relative high frequency of ileus due to adhesions during later life was observed. 

One of the advantages of the Gore-Tex dual mesh® is that it gives the possibility to obtain tension on the fascial edges avoiding lateralization, which happens with the Silastic silo method. Moreover, when secondary fascial closure is impossible, the mesh may be left in situ which help creating strong fibrous tissue beneath the mesh when it may be removed allowing self-epithelialization or skin grafting. The disadvantage with the mesh is that it is not translucent, and therefore it is not possible to observe the intestinal loops for ischemia. However, this complication was not seen in any of our patients. 

To date the use of GDM as a prosthetic material in closure of congenital abdominal wall defects has been described in smaller series only. Rahn et al. [5] compared the use of a dura patch versus GDM in the management of congenital wall defects. In their series, four children were successfully treated with GDM; a smooth underlying pseudo-membrane was formed, that provided a stable covering to the eviscerated viscera, in all four children followed by secondary abdominal wall reconstruction at the age of 20-30 months. Stringel [9] and Willis [10] evaluated the clinical utility of Gore-Tex mesh for the repair of neonatal congenital abdominal wall defects in three and ten infants respectively. The Gore-Tex meshes used were from W. L. Gore and associates, Flagstaff, Arizona, USA, but it was not specified whether it was GDM. In both of series, frequent local infection and mesh removal were reported. However, both authors concluded that Gore-Tex was a useful patch for closure of congenital abdominal wall defects and a safe alternative in cases where primary closure is not feasible. Rijhwani [6] et al described similar results in three children treated by the use of a Gore-Tex mesh requiring mesh removal due to mesh-related infections. 

Rijhwani et al. [6] reported frequent late operations due to ventral (60%) and inguinal (30%) hernias in infants operated by staged closure, without specifying whether Gore-Tex or other silo-material was used. In our series, three children (10%) were operated for ventral hernia; only one child (4%) who had a successful secondary facial closure needed surgery for a ventral hernia. This indicates that closure of the abdomen by bridging the defect with a mesh until final apposition of the fascial edges and skin can be obtained creates a stable abdominal wall in the vast majority of cases. A disadvantage related to the use of the mesh might be the need of repeated surgical procedures in general anesthesia, but it is worthwhile to mention that no procedure-related complications were observed. The relative high in-hospital mortality observed in our study was mainly related to concomitant malformations and immaturity. This is in accordance with previous studies [11-14].

The limitations in the present study are its retrospective nature and a less significant study model but we present a cohort of 34 children, which to our knowledge is the largest described cohort of children with congenital abdominal wall defect operated by the using of a GDM.

## CONCLUSION

When primary closure is not possible, staged closure with GDM is a safe alternative creating a stable abdominal wall with few mesh-related neonatal complications. Clinicians and parents should be aware of an increased risk of ileus in later life with this mesh.

## Footnotes

**Source of Support:** None

**Conflict of Interest:** None
